# 1-Formyl-*t*-3,*t*-5-dimethyl-*r*-2,*c*-6-diphenyl­piperidin-4-one

**DOI:** 10.1107/S1600536810018490

**Published:** 2010-05-29

**Authors:** K. Ravichandran, P. Ramesh, P. Sakthivel, S. Ponnuswamy, M. N. Ponnuswamy

**Affiliations:** a Centre of Advanced Study in Crystallography and Biophysics, University of Madras, Guindy Campus, Chennai 600 025, India; b Department of Chemistry, Government Arts College (Autonomous), Coimbatore 641 018, India

## Abstract

In the title compound, C_20_H_21_NO_2_, the piperidine ring adopts a distorted boat conformation. The dihedral angle between the two phenyl rings is 61.33 (18)°. In the crystal, inter­molecular C—H⋯O inter­actions link the mol­ecules into zigzag *C*(5) chains running parallel to [100].

## Related literature

For general background to piperidine derivatives, see: Perumal *et al.* (2001 ▶); Dimmock *et al.* (2001 ▶). For asymmetry parameters, see: Nardelli (1983 ▶). For puckering parameters, see: Cremer & Pople (1975 ▶). For hydrogen-bond motifs, see: Bernstein *et al.* (1995 ▶). For the synthesis, see: Jeyaraman *et al.* (1999 ▶).
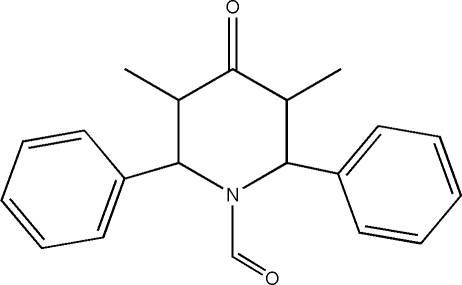


## Experimental

### 

#### Crystal data

C_20_H_21_NO_2_
*M* *_r_* = 307.38
Orthorhombic, 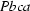

*a* = 7.4303 (4) Å
*b* = 15.3567 (6) Å
*c* = 29.6732 (13) Å
*V* = 3385.9 (3) Å^3^
*Z* = 8
Mo *K*α radiation
μ = 0.08 mm^−1^
*T* = 292 K
0.22 × 0.19 × 0.16 mm

#### Data collection

Bruker SMART APEXII area-detector diffractometer
Absorption correction: multi-scan (*SADABS*; Bruker, 2008 ▶) *T*_min_ = 0.983, *T*_max_ = 0.988
16882 measured reflections
4198 independent reflections
2097 reflections with *I* > 2σ(*I*)
*R*_int_ = 0.030

#### Refinement

*R*[*F* ^2^ > 2σ(*F* ^2^)] = 0.059
*wR*(*F* ^2^) = 0.194
*S* = 1.02
4198 reflections
213 parameters
25 restraints
H atoms treated by a mixture of independent and constrained refinement
Δρ_max_ = 0.37 e Å^−3^
Δρ_min_ = −0.23 e Å^−3^

### 

Data collection: *APEX2* (Bruker, 2008 ▶); cell refinement: *SAINT* (Bruker, 2008 ▶); data reduction: *SAINT*; program(s) used to solve structure: *SHELXS97* (Sheldrick, 2008 ▶); program(s) used to refine structure: *SHELXL97* (Sheldrick, 2008 ▶); molecular graphics: *ORTEP-3* (Farrugia, 1997 ▶); software used to prepare material for publication: *SHELXL97* and *PLATON* (Spek, 2009 ▶).

## Supplementary Material

Crystal structure: contains datablocks global, I. DOI: 10.1107/S1600536810018490/ci5087sup1.cif

Structure factors: contains datablocks I. DOI: 10.1107/S1600536810018490/ci5087Isup2.hkl

Additional supplementary materials:  crystallographic information; 3D view; checkCIF report

## Figures and Tables

**Table 1:** Hydrogen-bond geometry (Å, °)

*D*—H⋯*A*	*D*—H	H⋯*A*	*D*⋯*A*	*D*—H⋯*A*
C6—H6⋯O2^i^	0.98	2.48	3.398 (3)	156

Symmetry code: (i) 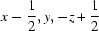.

**Fig. 1.**
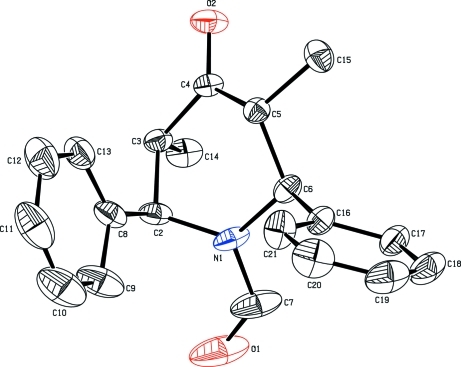
The molecular structure of the title compound. Displacement ellipsoids are drawn at the 30% probability level. H atoms have been omitted for clarity.

**Fig. 2.**
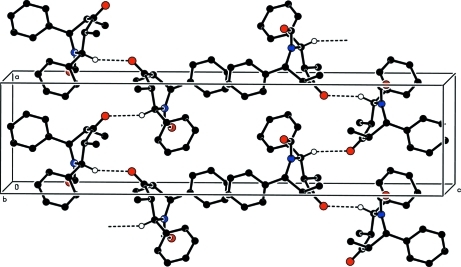
The crystal packing of the molecules viewed down the b axis. H atoms not involved in hydrogen bonding have been omitted for clarity.

## supplementary crystallographic information

### Comment

Piperidine derivatives are valued heterocyclic compounds in the field of medicinal chemistry. Piperidin-4-ones are reported to possess analgesic, anti-inflammatory, central nervous system (CNS), local anaesthetic, anticancer and antimicrobial activities (Perumal *et al.*,  2001; Dimmock *et al.*, 2001). The crystallographic study of the title compound has been carried out to establish the molecular structure.

In the title molecule (Fig. 1), the piperidine ring adopts a distorted boat conformation; the puckering (Cremer & Pople, 1975) and asymmetry parameters (Nardelli, 1983) are: q_2_ = 0.637 (3) Å, q_3_ = -0.052 (3) Å, φ_2_ = 245.5 (2)° and Δ_s_(C2 & C5) = 3.5 (2)°. The C8—C13 and C16—C21 phenyl rings are in axial [C4—C3—C2—C8 = 75.6 (3)°] and equatorial [C4—C5—C6—C16 = -166.8 (2)°] orientations, respectively. The methyl groups attached to atoms C3 and C5 of the piperidine ring are in axial and equatorial orientations [N1—C2—C3—C14 = 69.9 (2)° and N1—C6—C5—C15 = -168.1 (2)°]. The sum of bond angles around atom N1 (359.5°) of the piperidine ring is in accordance with sp^2^ hybridization.

Atom C6 at (x, y, z) acts as a hydrogen-bond donor to atom O2 of the molecule at (x-1/2, y, 1/2-z) forming a zigzag C(5) chain (Bernstein *et al.*, 1995) running along the *a* axis, as shown in Fig. 2.

### Experimental

An ice-cold solution of acetic-formic anhydride prepared from acetic anhydride (10 ml) and 85% formic acid (5 ml) was added slowly to a cold solution of r-2,c-6-diphenyl-t-3,t-5-dimethyl piperidin-4-one (1.395 g, 5 mmol) in benzene (30 ml). The reaction mixture was stirred at room temperature for 5 h. The organic layer was separated, dried over anhydrous Na_2_SO_4_ and concentrated. The resulting mass was purified and crystallized from benzene- petroleum ether (333-335 K) in the ratio 1:1 (Jeyaraman *et al.*, 1999).

### Refinement

Atom H7 was located in a difference map and its positional parameters were refined. The remaining H atoms were positioned geometrically (C–H =0.93-0.98 Å, ) and allowed to ride on their parent atoms, with 1.5U_eq_(C) for methyl and 1.2 U_eq_(C) for other H atoms. The U^ij^ parameters of atoms O1, C7, C10 and C11 were restrained to an approximate isotropic behaviour.

### Crystal data

**Table:**

C_20_H_21_NO_2_	*F*(000) = 1312
*M**_r_* = 307.38	*D*_x_ = 1.206 Mg m^−^^3^
Orthorhombic, *P**b**c**a*	Mo *K*α radiation, λ = 0.71073 Å
Hall symbol: -P 2ac 2ab	Cell parameters from 1246 reflections
*a* = 7.4303 (4) Å	θ = 1.4–28.4°
*b* = 15.3567 (6) Å	µ = 0.08 mm^−^^1^
*c* = 29.6732 (13) Å	*T* = 292 K
*V* = 3385.9 (3) Å^3^	Block, colourless
*Z* = 8	0.22 × 0.19 × 0.16 mm

### Data collection

**Table:**

Bruker SMART APEXII area-detector diffractometer	4198 independent reflections
Radiation source: fine-focus sealed tube	2097 reflections with *I* > 2σ(*I*)
graphite	*R*_int_ = 0.030
ω and φ scans	θ_max_ = 28.4°, θ_min_ = 1.4°
Absorption correction: multi-scan (*SADABS*; Bruker, 2008)	*h* = −9→6
*T*_min_ = 0.983, *T*_max_ = 0.988	*k* = −20→18
16882 measured reflections	*l* = −39→38

### Refinement

**Table:**

Refinement on *F*^2^	Primary atom site location: structure-invariant direct methods
Least-squares matrix: full	Secondary atom site location: difference Fourier map
*R*[*F*^2^ > 2σ(*F*^2^)] = 0.059	Hydrogen site location: inferred from neighbouring sites
*wR*(*F*^2^) = 0.194	H atoms treated by a mixture of independent and constrained refinement
*S* = 1.01	*w* = 1/[σ^2^(*F*_o_^2^) + (0.0741*P*)^2^ + 1.1948*P*] where *P* = (*F*_o_^2^ + 2*F*_c_^2^)/3
4198 reflections	(Δ/σ)_max_ = 0.001
213 parameters	Δρ_max_ = 0.37 e Å^−^^3^
25 restraints	Δρ_min_ = −0.22 e Å^−^^3^

### Special details

**Table:**

Geometry. All esds (except the esd in the dihedral angle between two l.s. planes) are estimated using the full covariance matrix. The cell esds are taken into account individually in the estimation of esds in distances, angles and torsion angles; correlations between esds in cell parameters are only used when they are defined by crystal symmetry. An approximate (isotropic) treatment of cell esds is used for estimating esds involving l.s. planes.
Refinement. Refinement of *F*^2^ against ALL reflections. The weighted *R*-factor wR and goodness of fit *S* are based on *F*^2^, conventional *R*-factors *R* are based on *F*, with *F* set to zero for negative *F*^2^. The threshold expression of *F*^2^ > σ(*F*^2^) is used only for calculating *R*-factors(gt) etc. and is not relevant to the choice of reflections for refinement. *R*-factors based on *F*^2^ are statistically about twice as large as those based on *F*, and *R*- factors based on ALL data will be even larger.

### Fractional atomic coordinates and isotropic or equivalent isotropic displacement parameters (Å^2^)

**Table:**

	*x*	*y*	*z*	*U*_iso_*/*U*_eq_	
O1	0.0192 (4)	0.2060 (2)	0.13459 (14)	0.1697 (16)	
O2	0.6395 (3)	0.38855 (13)	0.21978 (6)	0.0841 (6)	
N1	0.2046 (3)	0.31856 (13)	0.14813 (8)	0.0690 (6)	
C2	0.3721 (3)	0.27494 (15)	0.13546 (9)	0.0645 (7)	
H2	0.3426	0.2132	0.1317	0.077*	
C3	0.5024 (3)	0.28034 (15)	0.17521 (8)	0.0565 (6)	
H3	0.6204	0.2596	0.1651	0.068*	
C4	0.5235 (3)	0.37170 (15)	0.19238 (8)	0.0563 (6)	
C5	0.3986 (3)	0.44063 (14)	0.17429 (8)	0.0535 (6)	
H5	0.4444	0.4570	0.1445	0.064*	
C6	0.2059 (3)	0.40703 (16)	0.16730 (8)	0.0586 (6)	
H6	0.1498	0.4034	0.1971	0.070*	
C7	0.0458 (5)	0.2769 (3)	0.14840 (17)	0.1186 (15)	
C8	0.4415 (4)	0.30662 (16)	0.09006 (9)	0.0715 (8)	
C9	0.3332 (6)	0.2922 (2)	0.05318 (12)	0.1179 (14)	
H9	0.2243	0.2632	0.0569	0.141*	
C10	0.3834 (9)	0.3199 (3)	0.01118 (15)	0.1455 (18)	
H10	0.3106	0.3078	−0.0135	0.175*	
C11	0.5382 (9)	0.3649 (3)	0.00538 (13)	0.1369 (17)	
H11	0.5679	0.3869	−0.0229	0.164*	
C12	0.6510 (6)	0.3778 (3)	0.04115 (13)	0.1199 (14)	
H12	0.7601	0.4065	0.0371	0.144*	
C13	0.6019 (5)	0.3479 (2)	0.08344 (10)	0.0854 (9)	
H13	0.6794	0.3560	0.1077	0.103*	
C14	0.4401 (4)	0.22289 (18)	0.21443 (10)	0.0781 (8)	
H14A	0.5236	0.2281	0.2390	0.117*	
H14B	0.4348	0.1633	0.2047	0.117*	
H14C	0.3229	0.2413	0.2242	0.117*	
C15	0.3989 (4)	0.52323 (17)	0.20290 (10)	0.0779 (8)	
H15A	0.3343	0.5128	0.2304	0.117*	
H15B	0.3418	0.5695	0.1865	0.117*	
H15C	0.5207	0.5393	0.2098	0.117*	
C16	0.0934 (3)	0.46917 (17)	0.13948 (9)	0.0627 (6)	
C17	−0.0611 (4)	0.50498 (19)	0.15754 (11)	0.0781 (8)	
H17	−0.0950	0.4895	0.1866	0.094*	
C18	−0.1656 (4)	0.5623 (2)	0.13407 (17)	0.1041 (12)	
H18	−0.2686	0.5855	0.1472	0.125*	
C19	−0.1192 (6)	0.5849 (2)	0.09201 (18)	0.1146 (14)	
H19	−0.1902	0.6241	0.0760	0.138*	
C20	0.0328 (6)	0.5506 (2)	0.07227 (12)	0.1109 (12)	
H20	0.0638	0.5662	0.0430	0.133*	
C21	0.1400 (5)	0.4924 (2)	0.09632 (10)	0.0881 (9)	
H21	0.2432	0.4694	0.0832	0.106*	
H7	−0.076 (5)	0.287 (2)	0.1562 (11)	0.106*	

### Atomic displacement parameters (Å^2^)

**Table:**

	*U*^11^	*U*^22^	*U*^33^	*U*^12^	*U*^13^	*U*^23^
O1	0.113 (2)	0.099 (2)	0.298 (4)	−0.0455 (18)	−0.089 (2)	0.053 (2)
O2	0.0795 (13)	0.0781 (13)	0.0947 (14)	−0.0123 (10)	−0.0379 (11)	−0.0045 (10)
N1	0.0443 (11)	0.0572 (12)	0.1055 (16)	−0.0146 (10)	−0.0220 (11)	0.0253 (11)
C2	0.0677 (16)	0.0427 (12)	0.0832 (17)	−0.0081 (12)	−0.0297 (13)	0.0010 (12)
C3	0.0484 (12)	0.0515 (13)	0.0695 (14)	−0.0012 (11)	−0.0139 (11)	0.0017 (11)
C4	0.0486 (12)	0.0571 (14)	0.0631 (14)	−0.0099 (11)	−0.0099 (11)	0.0045 (11)
C5	0.0527 (12)	0.0480 (12)	0.0597 (13)	−0.0046 (10)	0.0025 (10)	0.0044 (10)
C6	0.0474 (12)	0.0678 (15)	0.0606 (13)	0.0004 (11)	0.0032 (10)	0.0163 (12)
C7	0.085 (2)	0.078 (2)	0.193 (4)	−0.038 (2)	−0.057 (3)	0.057 (2)
C8	0.096 (2)	0.0484 (13)	0.0705 (17)	0.0024 (14)	−0.0257 (15)	−0.0083 (12)
C9	0.172 (4)	0.095 (2)	0.087 (2)	−0.018 (2)	−0.062 (2)	0.0030 (19)
C10	0.222 (5)	0.127 (4)	0.087 (3)	−0.001 (4)	−0.060 (3)	−0.003 (2)
C11	0.222 (5)	0.120 (3)	0.069 (2)	0.018 (3)	0.006 (3)	−0.008 (2)
C12	0.153 (4)	0.123 (3)	0.083 (2)	−0.004 (3)	0.030 (3)	−0.015 (2)
C13	0.101 (2)	0.089 (2)	0.0661 (17)	−0.0029 (19)	0.0057 (16)	−0.0164 (15)
C14	0.0777 (18)	0.0679 (17)	0.0887 (19)	−0.0093 (14)	−0.0188 (15)	0.0189 (14)
C15	0.094 (2)	0.0595 (16)	0.0801 (18)	0.0007 (15)	0.0063 (16)	−0.0074 (14)
C16	0.0525 (13)	0.0661 (15)	0.0696 (15)	0.0061 (12)	−0.0013 (11)	0.0106 (12)
C17	0.0534 (15)	0.0730 (18)	0.108 (2)	0.0041 (14)	0.0022 (15)	0.0017 (16)
C18	0.0604 (18)	0.070 (2)	0.182 (4)	0.0121 (16)	−0.019 (2)	0.005 (2)
C19	0.095 (3)	0.075 (2)	0.174 (4)	0.011 (2)	−0.054 (3)	0.029 (3)
C20	0.137 (3)	0.098 (3)	0.097 (2)	0.008 (3)	−0.023 (2)	0.037 (2)
C21	0.097 (2)	0.089 (2)	0.0784 (18)	0.0259 (17)	0.0042 (16)	0.0265 (16)

### Geometric parameters (Å, °)

**Table:**

O1—C7	1.180 (5)	C10—H10	0.93
O2—C4	1.213 (3)	C11—C12	1.367 (6)
N1—C7	1.342 (4)	C11—H11	0.93
N1—C2	1.462 (3)	C12—C13	1.385 (4)
N1—C6	1.473 (3)	C12—H12	0.93
C2—C8	1.522 (4)	C13—H13	0.93
C2—C3	1.528 (3)	C14—H14A	0.96
C2—H2	0.98	C14—H14B	0.96
C3—C4	1.501 (3)	C14—H14C	0.96
C3—C14	1.532 (3)	C15—H15A	0.96
C3—H3	0.98	C15—H15B	0.96
C4—C5	1.507 (3)	C15—H15C	0.96
C5—C15	1.526 (3)	C16—C21	1.374 (4)
C5—C6	1.536 (3)	C16—C17	1.381 (4)
C5—H5	0.98	C17—C18	1.365 (4)
C6—C16	1.513 (3)	C17—H17	0.93
C6—H6	0.98	C18—C19	1.341 (5)
C7—H7	0.95 (4)	C18—H18	0.93
C8—C13	1.363 (4)	C19—C20	1.378 (5)
C8—C9	1.376 (4)	C19—H19	0.93
C9—C10	1.369 (6)	C20—C21	1.393 (4)
C9—H9	0.93	C20—H20	0.93
C10—C11	1.353 (7)	C21—H21	0.93
			
C7—N1—C2	122.1 (3)	C9—C10—H10	119.8
C7—N1—C6	116.3 (3)	C10—C11—C12	119.8 (4)
C2—N1—C6	121.11 (17)	C10—C11—H11	120.1
N1—C2—C8	111.7 (2)	C12—C11—H11	120.1
N1—C2—C3	108.4 (2)	C11—C12—C13	119.6 (4)
C8—C2—C3	116.8 (2)	C11—C12—H12	120.2
N1—C2—H2	106.4	C13—C12—H12	120.2
C8—C2—H2	106.4	C8—C13—C12	121.0 (3)
C3—C2—H2	106.4	C8—C13—H13	119.5
C4—C3—C2	112.28 (19)	C12—C13—H13	119.5
C4—C3—C14	108.2 (2)	C3—C14—H14A	109.5
C2—C3—C14	111.3 (2)	C3—C14—H14B	109.5
C4—C3—H3	108.3	H14A—C14—H14B	109.5
C2—C3—H3	108.3	C3—C14—H14C	109.5
C14—C3—H3	108.3	H14A—C14—H14C	109.5
O2—C4—C3	120.1 (2)	H14B—C14—H14C	109.5
O2—C4—C5	121.8 (2)	C5—C15—H15A	109.5
C3—C4—C5	118.12 (19)	C5—C15—H15B	109.5
C4—C5—C15	112.6 (2)	H15A—C15—H15B	109.5
C4—C5—C6	112.73 (18)	C5—C15—H15C	109.5
C15—C5—C6	110.8 (2)	H15A—C15—H15C	109.5
C4—C5—H5	106.7	H15B—C15—H15C	109.5
C15—C5—H5	106.7	C21—C16—C17	117.9 (3)
C6—C5—H5	106.7	C21—C16—C6	122.2 (2)
N1—C6—C16	111.6 (2)	C17—C16—C6	119.9 (2)
N1—C6—C5	111.58 (19)	C18—C17—C16	122.1 (3)
C16—C6—C5	112.11 (19)	C18—C17—H17	118.9
N1—C6—H6	107.1	C16—C17—H17	118.9
C16—C6—H6	107.1	C19—C18—C17	119.7 (3)
C5—C6—H6	107.1	C19—C18—H18	120.1
O1—C7—N1	125.8 (5)	C17—C18—H18	120.1
O1—C7—H7	94 (2)	C18—C19—C20	120.5 (3)
N1—C7—H7	140 (2)	C18—C19—H19	119.8
C13—C8—C9	118.1 (3)	C20—C19—H19	119.8
C13—C8—C2	124.9 (2)	C19—C20—C21	119.8 (3)
C9—C8—C2	117.0 (3)	C19—C20—H20	120.1
C10—C9—C8	121.0 (4)	C21—C20—H20	120.1
C10—C9—H9	119.5	C16—C21—C20	120.0 (3)
C8—C9—H9	119.5	C16—C21—H21	120.0
C11—C10—C9	120.4 (4)	C20—C21—H21	120.0
C11—C10—H10	119.8		
			
C7—N1—C2—C8	109.2 (3)	C6—N1—C7—O1	−179.9 (4)
C6—N1—C2—C8	−79.4 (3)	N1—C2—C8—C13	117.9 (3)
C7—N1—C2—C3	−120.7 (3)	C3—C2—C8—C13	−7.7 (4)
C6—N1—C2—C3	50.6 (3)	N1—C2—C8—C9	−62.1 (3)
N1—C2—C3—C4	−51.5 (3)	C3—C2—C8—C9	172.3 (3)
C8—C2—C3—C4	75.6 (3)	C13—C8—C9—C10	−1.2 (5)
N1—C2—C3—C14	69.9 (2)	C2—C8—C9—C10	178.8 (4)
C8—C2—C3—C14	−162.9 (2)	C8—C9—C10—C11	−2.3 (7)
C2—C3—C4—O2	−170.3 (2)	C9—C10—C11—C12	4.3 (8)
C14—C3—C4—O2	66.4 (3)	C10—C11—C12—C13	−2.7 (7)
C2—C3—C4—C5	9.2 (3)	C9—C8—C13—C12	2.7 (5)
C14—C3—C4—C5	−114.1 (2)	C2—C8—C13—C12	−177.3 (3)
O2—C4—C5—C15	−16.0 (3)	C11—C12—C13—C8	−0.8 (6)
C3—C4—C5—C15	164.5 (2)	N1—C6—C16—C21	−66.4 (3)
O2—C4—C5—C6	−142.4 (2)	C5—C6—C16—C21	59.5 (3)
C3—C4—C5—C6	38.2 (3)	N1—C6—C16—C17	114.1 (3)
C7—N1—C6—C16	−65.6 (3)	C5—C6—C16—C17	−120.0 (3)
C2—N1—C6—C16	122.6 (2)	C21—C16—C17—C18	−0.5 (4)
C7—N1—C6—C5	168.1 (3)	C6—C16—C17—C18	179.0 (3)
C2—N1—C6—C5	−3.7 (3)	C16—C17—C18—C19	0.3 (5)
C4—C5—C6—N1	−40.9 (3)	C17—C18—C19—C20	0.2 (6)
C15—C5—C6—N1	−168.1 (2)	C18—C19—C20—C21	−0.5 (6)
C4—C5—C6—C16	−166.8 (2)	C17—C16—C21—C20	0.1 (5)
C15—C5—C6—C16	65.9 (3)	C6—C16—C21—C20	−179.4 (3)
C2—N1—C7—O1	−8.2 (6)	C19—C20—C21—C16	0.4 (5)

### Hydrogen-bond geometry (Å, °)

**Table:**

*D*—H···*A*	*D*—H	H···*A*	*D*···*A*	*D*—H···*A*
C6—H6···O2^i^	0.98	2.48	3.398 (3)	156

Symmetry codes: (i) *x*−1/2, *y*, −*z*+1/2.
